# Longitudinal trajectories of branched chain amino acids through young adulthood and diabetes in later life

**DOI:** 10.1172/jci.insight.166956

**Published:** 2023-04-24

**Authors:** Konrad T. Sawicki, Hongyan Ning, Norrina B. Allen, Mercedes R. Carnethon, Amisha Wallia, James D. Otvos, Issam Ben-Sahra, Elizabeth M. McNally, Janet K. Snell-Bergeon, John T. Wilkins

**Affiliations:** 1 Division of Cardiology, Department of Medicine;; 2 Department of Preventive Medicine; and; 3 Division of Endocrinology, Metabolism, and Molecular Medicine, Department of Medicine, Northwestern University Feinberg School of Medicine, Chicago, Illinois, USA.; 4 Laboratory Corporation of America Holdings (LabCorp), Morrisville, North Carolina, USA.; 5 Department of Biochemistry and Molecular Genetics and; 6 Center for Genetic Medicine, Northwestern University Feinberg School of Medicine, Chicago, Illinois, USA.; 7 Barbara Davis Center for Diabetes, University of Colorado Anschutz Medical Campus, Aurora, Colorado, USA.

**Keywords:** Endocrinology, Metabolism, Diabetes, Epidemiology

## Abstract

**BACKGROUND:**

Elevated circulating branched chain amino acids (BCAAs), measured at a single time point in middle life, are strongly associated with an increased risk of developing type 2 diabetes mellitus (DM). However, the longitudinal patterns of change in BCAAs through young adulthood and their association with DM in later life are unknown.

**METHODS:**

We serially measured BCAAs over 28 years in the Coronary Artery Risk Development in Young Adults (CARDIA) study, a prospective cohort of apparently healthy Black and White young adults at baseline. Trajectories of circulating BCAA concentrations from years 2–30 (for prevalent DM) or years 2–20 (for incident DM) were determined by latent class modeling.

**RESULTS:**

Among 3,081 apparently healthy young adults, trajectory analysis from years 2–30 revealed 3 distinct BCAA trajectory groups: low-stable (*n* = 1,427), moderate-stable (*n* = 1,384), and high-increasing (*n* = 270) groups. Male sex, higher body mass index, and higher atherogenic lipid fractions were more common in the moderate-stable and high-increasing groups. Higher risk of prevalent DM was associated with the moderate-stable (OR = 2.59, 95% CI: 1.90–3.55) and high-increasing (OR = 6.03, 95% CI: 3.86–9.43) BCAA trajectory groups in adjusted models. A separate trajectory group analysis from years 2–20 for incident DM after year 20 showed that moderate-stable and high-increasing trajectory groups were also significantly associated with higher risk of incident DM, after adjustment for clinical variables and glucose levels.

**CONCLUSION:**

BCAA levels track over a 28-year span in most young adults, but serial clinical metabolomic measurements identify subpopulations with rising levels associated with high risk of DM in later life.

**FUNDING:**

This research was supported by the NIH, under grants R01 HL146844 (JTW) and T32 HL069771 (MRC). The CARDIA study is conducted and supported by the NIH National Heart, Lung, and Blood Institute in collaboration with the University of Alabama at Birmingham (HHSN268201800005I and HHSN268201800007I), Northwestern University (HHSN268201800003I), the University of Minnesota (HHSN268201800006I), and Kaiser Foundation Research Institute (HHSN268201800004I).

## Graphical abstract

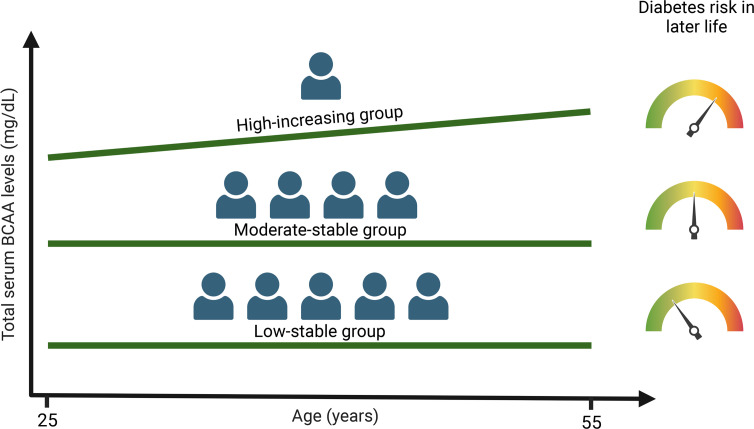

## Introduction

In type 2 diabetes mellitus (DM), significant pancreatic β cell dysfunction occurs before the onset of hyperglycemia (1). The ability to identify individuals in early subclinical stages of DM is particularly important because proven preventive therapies exist and end-organ complications accumulate over time (2, 3).

Metabolomic profiling offers the potential to reveal metabolites and metabolic pathways relevant to the pathophysiology of insulin resistance and improve the identification of those at risk for DM. A prominent amino acid signature of circulating branched chain amino acids (BCAAs) in normoglycemic middle-aged adults is positively correlated with future development of DM (4). However, the vast majority of prior circulating BCAA studies have focused on middle-aged, high-risk adults largely of European descent (4, 5). The association of circulating BCAA profiles and the development of DM in younger, low-risk adults, particularly of non-European ancestry, is not well understood (6).

Interventions like gastric bypass surgery lower plasma BCAA levels, suggesting that BCAA levels are dynamic and modifiable (7). Recently, plasma BCAA levels were measured at 2 time points approximately 10 years apart among middle-aged, White women in the Nurses’ Health Study (8). However, no study to our knowledge has performed longitudinal serial profiling of BCAAs to assess the natural variation in these metabolites over the human life span in a diverse cohort. Understanding the longitudinal patterns of change in BCAAs in a diverse sample of apparently healthy young adults and their associations with DM in later life may enhance our understanding of the pathophysiology of insulin resistance and guide preventive interventions in select young individuals at high risk for developing DM.

Here, we use a nuclear magnetic resonance–based (NMR-based) metabolite profiling platform to serially measure total plasma BCAAs in the Coronary Artery Risk Development in Young Adults (CARDIA) study, a diverse cohort of young Black and White adults followed longitudinally over the course of more than 35 years. We hypothesized that plasma BCAA levels increase over time across young and middle adulthood in certain individuals and that an increasing BCAA trajectory is independently associated with higher risk of prevalent and incident DM in later life.

## Results

**Figure 1:**
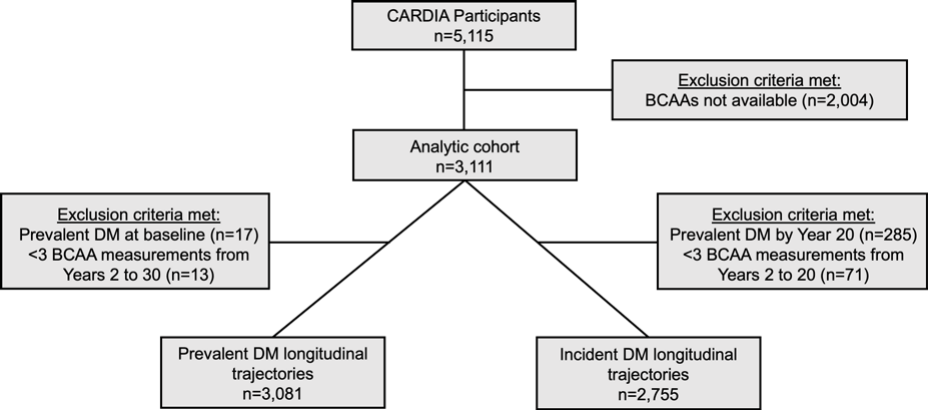
Flow diagram for study inclusion. BCAA, branched chain amino acid; CARDIA, Coronary Artery Risk Development in Young Adults; DM, diabetes mellitus.

Of the 5,115 participants in CARDIA, there were 3,111 participants with available longitudinal BCAA measurements included in this analysis (Figure 1). At the baseline year 2 examination, the mean age of the analytic cohort was 27.2 years, 44.2% were men, 44.7% were Black, the mean body mass index (BMI) was 25.1 kg/m^2^, and the mean fasting blood glucose was 85.3 mg/dL. At the year 2 examination, 29 participants (1.0%) had prevalent DM; by the year 30 examination, 422 participants (16.5%) had prevalent DM (Supplemental Table 1; supplemental material available online with this article; https://doi.org/10.1172/jci.insight.166956DS1). Men had higher circulating BCAA levels compared with women, and baseline year 2 participant characteristics were similar in sex-stratified analyses across quartiles of BCAAs (Supplemental Table 2).

Detailed characteristics of participants included and excluded from this analysis are available in Supplemental Table 3. Compared with participants in the final analytic cohort, CARDIA participants who were excluded from this analysis had higher triglyceride levels and more likely self-identified as Black and current smokers at the baseline year 2 examination.

### Longitudinal trajectories of circulating BCAAs from the year 2 to 30 exams.

**Figure 2:**
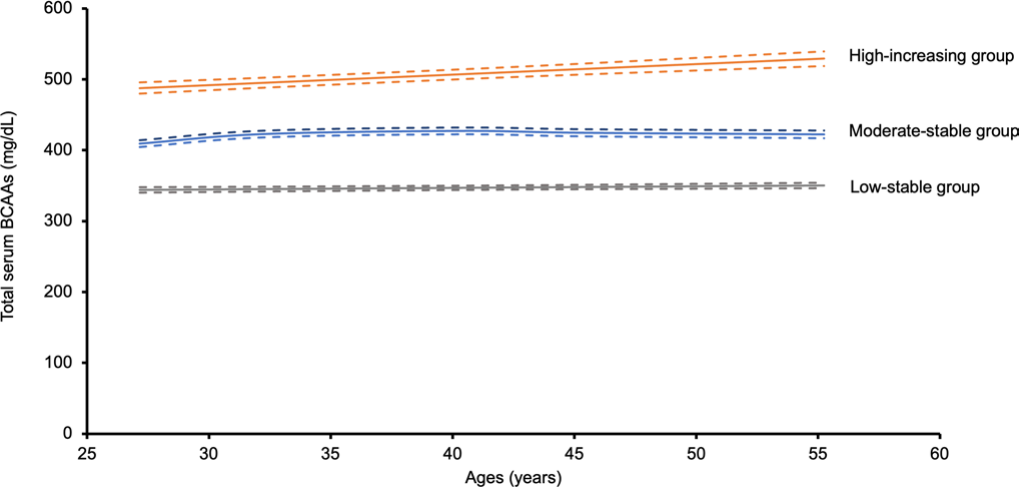
Longitudinal trajectories of BCAAs over a 28-year span. Of the 3,081 participants included for the prevalent DM longitudinal BCAA trajectory group modeling the year 2 to 30 examinations, there were 1,427 (46.3%) participants in the low-stable group, 1,384 (44.9%) participants in the moderate-stable group, and 270 (8.8%) participants in the high-increasing group. Dotted lines indicate 95% CIs of the trajectory model estimate. BCAA, branched chain amino acid.

Trajectory group modeling was performed for the 3,081 nondiabetic participants at baseline. Among these participants, we identified 3 longitudinal trajectory groups of total BCAAs from the year 2 to 30 examinations: 1,427 (46.3%) participants with low total BCAAs that remained low throughout follow-up (low-stable), 1,384 (44.9%) participants with moderate total BCAAs that remained stable (moderate-stable), and 270 (8.8%) participants with high total BCAAs that increased during follow-up (high-increasing) (Figure 2). Similar trajectories were observed for men and women in sex-stratified analyses. The annualized rate of change in mean total BCAA levels from year 2 to year 30 examinations by trajectory group was 0.5 mg/dL/y in the low-stable group, 0.9 mg/dL/y in the moderate-stable group, and 2.3 mg/dL/y in the high-increasing group (Supplemental Table 4).

The baseline characteristics of participants by BCAA trajectory group are presented in Table 1. Compared with the low-stable group, participants in the moderate-stable and high-increasing groups were more likely to be male. Average systolic and diastolic blood pressures, total cholesterol, low-density lipoprotein cholesterol (LDL-C), physical activity score, and BMI were higher among the moderate-stable and high-increasing groups compared with the low-stable group. High-density lipoprotein cholesterol (HDL-C) was lower among the moderate-stable and high-increasing groups as well. The prevalence of DM at year 30 was substantially higher among the moderate-stable (20.3%) and high-increasing (40.9%) groups compared with the low-stable trajectory group (7.8%).

### Association of longitudinal BCAA trajectories with prevalent DM at year 30.

In multivariable-adjusted models including baseline glucose, the moderate-stable and high-increasing BCAA trajectory groups were respectively associated with 2.6 and 6.0 significantly higher odds of prevalent DM at year 30 compared with the low-stable trajectory group (Table 2). Similar patterns of association were observed for men and women in sex-stratified analyses. After further adjustment for cumulative risk factors, including fasting blood glucose, the associations of the moderate-stable and high-increasing BCAA trajectory groups with prevalent DM at year 30 were attenuated (Table 3).

### Association of longitudinal BCAA trajectories with incident DM at year 30.

**Figure 3:**
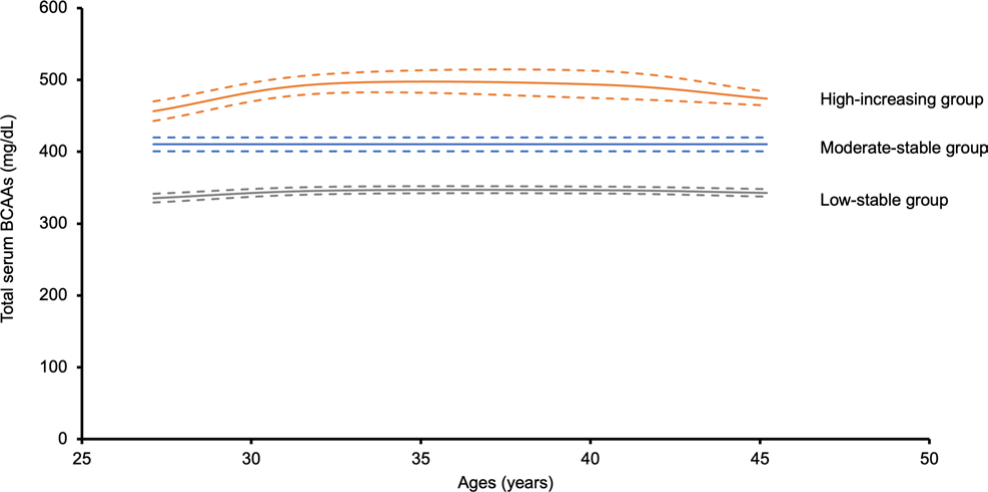
Longitudinal trajectories of BCAAs over an 18-year span. Of the 2,755 participants included for the incident DM longitudinal BCAA trajectory group modeling the year 2 to 20 examinations, there were 1,247 (45.3%) participants in the low-stable group, 1,196 (43.4%) participants in the moderate-stable group, and 312 (11.3%) participants in the high-increasing group. Dotted lines indicate 95% CIs of the trajectory model estimate. BCAA, branched chain amino acid.

To minimize the effect of bidirectional association between DM and elevated serum BCAA concentrations, we also assessed the association of year 2 to 20 BCAA trajectory groups with incident DM at the year 25 or 30 exams. Like the trajectory group model for prevalent DM, we identified 3 longitudinal trajectories of total BCAAs among these participants: 1,247 (45.3%) participants with low-stable total BCAA levels, 1,196 (43.4%) participants with moderate-stable total BCAA levels, and 312 (11.3%) participants with high-increasing total BCAA levels (Figure 3). The high-increasing group showed an initial increase in total BCAA levels, followed by a slight decline. The annualized rate of change in total BCAA levels from year 2 to year 30 examinations by trajectory group was 0.8 mg/dL in the low-stable group, 0.9 mg/dL in the moderate-stable group, and 1.9 mg/dL in the high-increasing group (Supplemental Table 5).

The baseline year 2 characteristics of participants by trajectory group for incident DM are shown in Table 4. Like the trajectory groups for prevalent DM, participants in the moderate-stable and high-increasing groups for incident DM were more likely to be men with higher systolic and diastolic blood pressures, total cholesterol, LDL-C, physical activity score, and BMI compared with the low-stable group. The incidence of DM after year 20 was higher among the moderate-stable (10.3%) and high-increasing groups (15.0%) compared with the low-stable group (4.4%).

The association between BCAA trajectory groups and the incidence of DM after year 20, after adjustment for demographic information and year 20 clinical covariates, including blood glucose, is shown in Table 5. The moderate-stable and high-increasing groups were respectively associated with 1.6 and 2.2 significantly higher odds of incident DM after year 20. There was no interaction by sex or race and ethnicity between the trajectory group and incident DM. In a separate model adjusting for cumulative risk factor burden, including fasting blood glucose from years 2 to 20, the association of the moderate-stable and high-increasing BCAA trajectory groups with increased incidence of DM after year 20 remained significant (Table 6).

## Discussion

In this longitudinal analysis of a comprehensively phenotyped biracial cohort of young adults, we provide the first description to our knowledge of BCAA patterns of change and associated characteristics over a 28-year span through young and mid-adulthood. First, we identified 3 distinct trajectories of total BCAAs: low-stable, moderate-stable, and high-increasing patterns. Second, higher BCAA blood trajectories were associated with male sex, higher blood pressure, and higher atherogenic lipid fractions in young adults. Third, higher trajectory patterns were strongly associated with incident DM after adjustment for serum glucose and the components of metabolic syndrome, suggesting that BCAA trajectories may capture aspects of DM risk not accounted for by traditional DM risk factors and hyperglycemia. In aggregate, these findings suggest that the metabolomic signatures of DM are present in young adulthood, they track in most individuals, and increasing BCAA blood concentration trajectories through young adulthood are predictors of future DM risk.

Interestingly, most young adults in the high-increasing BCAA trajectory group had fasting blood glucose and BMI values within the “normal” range (fasting blood glucose < 100 mg/dL, BMI < 25 mg/kg^2^) at the baseline year 2 examination. The higher DM risk observed in these groups suggests that elevated BCAAs are early markers of DM risk that precede the development of traditional risk factors in young adults or that the optimal levels of cardiometabolic risk factors may be lower than the thresholds currently used in clinical practice (9).

Previous descriptions of the relationship between total BCAAs and DM have relied on single baseline values of BCAAs (2–4, 6, 10). Through serial measurements, we identified 3 distinct longitudinal BCAA trajectory groups, which each carried varying degrees of risk for DM. Our findings demonstrate that elevated BCAAs in early adulthood and, importantly, their pattern of change over time are strongly associated with incident and prevalent DM in later life. Despite higher total levels of circulating BCAAs in men compared with women and in Black young adults compared with White young adults, we observed similar longitudinal trajectories and patterns of association between trajectory group and incident and prevalent DM in sex-stratified and race-stratified analyses. Additionally, the associations of BCAA trajectory with incident DM are independent of cumulative exposure to blood pressure, BMI, and glucose, indicating that BCAA trajectories reflect additional metabolic risk beyond traditionally assessed DM determinants. Additional studies are needed to determine if BCAA measurements (at one time point or multiple) enhance DM risk prediction beyond what can be predicted using parameters such as age, race, BMI, family history of DM, and metabolic measures (e.g., serum glucose).

Longitudinal measurements of BCAAs to identify trajectories of change may carry important clinical implications. Targeted metabolomics is increasingly being used in the clinical setting, such as NMR-based lipid derivative measurements (11, 12). It may soon be feasible for a clinician to assess trends in BCAA levels to identify apparently healthy young adults at high risk for developing DM. Such patients may benefit from earlier and more frequent screening for DM, as well as more aggressive risk factor control (e.g., weight loss, dietary interventions). Additionally, high-risk patients may particularly benefit from early initiation of metformin to prevent DM, as well as the renal and cardiovascular sequelae (13). Recent reports also suggest that antidiabetic medications can affect systemic BCAA metabolism. In patients with diabetes, empagliflozin led to higher plasma levels of the BCAA metabolite short-chain acylcarnitine (14). Future studies are needed to determine if the benefits of sodium/glucose co-transporter 2 inhibitors may be related to alterations in systemic or tissue-specific BCAA metabolism (15).

Although our observational study cannot demonstrate causality, our findings support the hypothesis that chronically elevated circulating BCAAs contribute to the pathogenesis of DM. Previous human Mendelian randomization studies have demonstrated that polymorphisms near the *PPM1K* gene (encoding branched chain alpha-keto acid dehydrogenase complex phosphatase) are associated with higher circulating BCAA levels and higher DM risk, suggesting that elevated circulating BCAAs are contributory to DM pathogenesis (16). However, the mechanisms underlying how elevations in BCAAs cause insulin resistance remain unclear. Further investigations into the mechanisms that lead to circulating BCAA elevations may provide novel therapeutic targets for the prevention of DM in high-risk young adults.

Our study has several strengths. First, the CARDIA study is a biracial cohort that has been extensively phenotyped over 9 examinations, which provides a potentially unique opportunity to describe patterns of BCAAs’ serum concentration change though young adulthood across race and sex. Second, NMR was used to provide absolute quantification of circulating BCAAs as opposed to liquid chromatography-mass spectrometry methods that provide relative concentrations. Third, DM at years 25 and 30 was comprehensively assessed using fasting blood glucose, oral glucose tolerance test, glycated hemoglobin (HbA1c), and antidiabetic medication history. Fourth, participants underwent in-person exams with high-quality assessment of metabolic characteristics that were used as covariates in our models. Fifth, we minimized reverse causality between DM and elevated BCAAs by performing a separate analysis of BCAA trajectories in nondiabetic individuals from years 2 to 20 and their association with incident DM at later exam dates, which remained significant (Tables 5 and 6).

The results of our study should also be put into the context of its limitations. The CARDIA study was designed to focus on the development of subclinical and clinical cardiovascular disease rather than the development of DM, and all possible DM parameters were not collected at all visits, particularly for the earlier examination dates. Additionally, our incidence model likely underestimates the true incidence of DM because of the exclusion of highly susceptible individuals who developed DM before the year 20 examination. However, only 285 out of 3,081 participants were excluded for the development of DM between years 2 and 20; thus, the effect of this exclusion is likely modest. While causality cannot be inferred from this longitudinal analysis alone, previous genetic studies strongly suggest that elevated BCAAs contribute to the development of DM. Although cardiorespiratory fitness was not included as a covariate because of lack of participants with available data, we controlled for physical activity in our logistic regression models. Although higher BCAA trajectory groups are strongly associated with DM development, additional studies are needed to determine the value of adding circulating BCAA levels to existing clinical DM risk prediction models. Finally, although nutritional BCAA intake was not specifically measured in CARDIA, we controlled for diet in logistic regression models using the HEI.

In summary, we identified 3 distinct longitudinal trajectories of BCAAs in a diverse cohort of apparently healthy young adults at baseline. We identified a group of young adults (approximately 10% of the cohort) who demonstrated a dynamic rise in BCAAs over 28 years who were at particularly high absolute risk for developing DM in later life. The trajectory of BCAAs from young adulthood to middle age was associated with incident DM independent of glucose level or cumulative risk factor burden. The longitudinal trajectory of BCAAs may provide additional insight into the risk of DM and provide early identification of high-risk young adults who may benefit most from aggressive risk factor modification.

## Methods

### CARDIA study sample.

The CARDIA study is a prospective cohort study of 5,115 participants that was designed to understand risk factors for the development of subclinical and overt cardiovascular disease. Black and White men and women aged 18–30 years were recruited from 1985–1986 across 4 urban US sites: Birmingham, Alabama; Chicago, Illinois; Minneapolis, Minnesota; and Oakland, California. Participants have been observed for over 30 years, with evaluation of demographic, clinical, laboratory, and imaging data, including height, weight, waist circumference, heart rate, blood pressure, lipid levels, glucose levels, dietary patterns, physical activity levels, smoking status, and education level. Participants in the CARDIA study have undergone in-person examinations at years 0, 2, 5, 7, 10, 15, 20, 25, and 30. Retention rates among surviving participants at each in-person examination have been high, at 91%, 86%, 81%, 79%, 74%, 72%, 72%, and 71%, respectively. Contact is maintained with participants via telephone, mail, or email every 6 months, with annual interim medical history ascertainment. Over the past 5 years, over 90% of the surviving cohort members have been directly contacted, and follow-up for vital status is virtually complete through related contacts and intermittent National Death Index searches.

Among 5,112 CARDIA study participants, inclusion criteria for this analysis included at least 3 BCAA measurements from years 2 to 30. There were 2,004 excluded because of insufficient serum or plasma sample availability. Of the remaining 3,111 participants with available BCAA measurements, 17 participants had prevalent DM at the baseline year 2 examination, and 13 participants were excluded for having inadequate NMR measurement. Thus, the final analytic cohort was composed of 3,081 participants (Figure 1). Participant characteristics by initial BCAA quartile used the year 7 BCAA levels if year 2 levels were not available. The number of participants with available BCAA measurements by examination year are shown in Supplemental Table 6.

### Measurement of traditional risk factors and lifestyle.

Age, race, and sex were determined via self-report. Height, weight, BMI (kg/m^2^), and waist circumference were measured with the participants in light clothing using standardized equipment. Detailed dietary data are available for years 0, 7, and 20. Dietary data obtained at the year 0 examination were used to represent year 2 dietary patterns. The CARDIA dietary data were obtained via interview that included a short questionnaire regarding general dietary practices followed by a comprehensive questionnaire about typical intake (food type, portion size, and frequency of consumption) of foods (17, 18). From these diet history data, the HEI at years 2 and 20 was calculated and used to represent overall dietary quality (19).

At each CARDIA examination, participants were given the interviewer-administered Physical Activity History Questionnaire. In brief, participants were asked about self-reported leisure time, the frequency of participation in 13 specific physical activity categories (8 of vigorous and 5 of moderate intensity) of recreational sports, structured exercise, home maintenance, and occupational activities during the preceding 12 months. Intensity of each activity was expressed as metabolic equivalents (20, 21).

Blood pressure was measured after 5 minutes of rest in the seated position using a standard sphygmomanometer. The means of the second and third systolic and fifth diastolic recordings were used. Alcohol intake, smoking habits, and educational attainment were determined with the use of standardized and validated questionnaires. Blood pressure– and cholesterol-lowering medication use was determined by self-report.

Plasma concentrations of total cholesterol and triglycerides were measured using a standard enzymatic assay. HDL-C was quantified after precipitation with dextral sulfate-magnesium chloride on an ABA 200 Biochromatic instrument (Abbott Laboratories). LDL-C was calculated using the Friedewald equation (22).

### Measurement of BCAAs.

After a 12-hour fast, blood was drawn from a vein in the antecubital fossa into a BD Vacutainer coated with EDTA for plasma. Serum and plasma samples were obtained and stored at –80°C for future analysis. We used targeted ^1^H-NMR to analyze previously banked serum samples. Analyses were performed at LabCorp on the high-throughput Vantera Clinical Analyzer platform. The validation of the use of NMR for quantification of BCAAs has been previously described (23, 24). In brief, the coefficients of variation for inter- and intra-assay precision for total BCAA, valine, leucine, and isoleucine ranged from 1.8%–6.0%, 1.7%–5.4%, 4.4%–9.1%, and 8.8%–21.3%, respectively. There was no significant difference in the ratio of individual BCAAs (leucine, isoleucine, valine) across time; thus, total BCAAs were used for analysis.

### Prevalent and incident diabetes definitions.

DM was defined as HbA1c ≥ 6.5% (years 20 and 25), 2-hour oral glucose tolerance test ≥ 200 mg/dL (available at years 10, 20, and 25), fasting blood glucose (obtained from NMR) ≥ 126 mg/dL, taking antidiabetic medications, or self-report of DM. Participants were categorized as having prevalent DM if any of the above DM definitions were positive at year 30. Incident DM was defined by meeting any of the criteria for DM listed above at either the year 25 or 30 exams.

Two separate BCAA trajectory models were performed among participants in CARDIA: prevalent DM model and incident DM model. The prevalent DM model included 3,081 CARDIA participants with 3 or more BCAA measurements during years 2 to 30. For the analysis that quantified the associations of longitudinal BCAA trajectories from years 2 to 30 with prevalent DM (*n* = 3,081), we excluded participants who did not have DM clinical information available at year 30 (*n* = 521). Thus, there were 2,560 CARDIA participants who met criteria to be included in the prevalent DM analysis (Figure 1).

The incident DM model included 2,755 CARDIA participants without DM by year 20 and with 3 or more BCAA measurements during years 2 to 20. For the analysis that quantified the associations between longitudinal BCAA trajectories from years 2 to 20 and incident DM after the year 20 exam, we excluded 285 CARDIA participants who developed DM by year 20. We excluded an additional 71 CARDIA participants who had fewer than 3 BCAA measurements from years 2 to 20 (3 or more measurements are needed to calculate a trajectory). Thus, there were 2,755 CARDIA participants who met criteria to be included in the incident DM analysis (Figure 1).

### Statistics.

We used latent class trajectory modeling to identify and categorize CARDIA participants based on patterns of longitudinal change in BCAAs from young to middle adulthood (25–29). The distinct patterns of longitudinal change in BCAA levels were determined using a customized SAS macro (PROC TRAJ). The censored normal model was used for continuous outcomes, and age at examinations was taken as the time scale. Bayesian information criteria and posterior probabilities were used to evaluate the fit of the most parsimonious trajectory model. In the final model, participants were classified into trajectory groups with excellent discrimination, and the posterior predicated probabilities across the trajectory groups ranged 0.85–0.89 (mean = 0.87). Longitudinal trajectory groups were defined as stable if the percentage change in absolute mean total BCAA levels over the period was <10% and increasing if the percentage change in mean absolute total BCAA levels over the period was >10%. There were no trajectory groups with decreasing BCAA levels over time at the group level. A threshold of >10% was chosen because it represents approximately 2-fold the coefficient of variation for total BCAAs.

Demographic characteristics and covariates for each of the BCAA trajectory groups were compared using Pearson’s χ^2^ test for categorical variables and ANOVA for continuous variables. Multivariable general linear models were used to evaluate the association of each trajectory group with prevalent or incident DM. The first model was unadjusted. The second model adjusted for the following clinical variables: age, sex, race and ethnicity, and education. The third model additionally adjusted for HDL-C, triglycerides, systolic blood pressure, waist circumference, smoking, antihypertensive medication treatment, HEI average index, and physical activity intensity. The fourth model additionally adjusted for fasting blood glucose as measured by NMR. Year 2 covariates were used for the prevalent DM analysis, and year 20 covariates were used for the incident DM analysis. The cumulative risk factor burden model adjusted for age, sex, race and ethnicity, education, and yearly averages (for characteristics not assessed at each examination) or cumulative values (for those characteristics assessed at all examinations, including glucose) of HDL-C, triglycerides, systolic blood pressure, waist circumference, antihypertensive medication treatment, smoking, physical activity intensity, HEI, and fasting blood glucose. There was no significant interaction of longitudinal BCAA trajectory groups and prevalent or incident DM with sex or race and ethnicity.

Two-tailed *P* values of less than 0.05 were considered statistically significant.

### Study approval.

The study was approved by the institutional review boards at each site: University of Alabama at Birmingham (Birmingham, Alabama, USA), Northwestern University (Chicago, Illinois, USA), University of Minnesota (Minneapolis, Minnesota, USA), and Kaiser Foundation Research Institute (Oakland, California, USA). All participants provided written informed consent.

## Author contributions

KTS designed the study, analyzed the data, and drafted the manuscript. HN analyzed the data and edited the manuscript. JDO acquired the data and edited the manuscript. NBA, MRC, AW, IBS, EMM, and JKSB analyzed the data and edited the manuscript for important intellectual content. JTW designed the study, acquired the data, analyzed the data, and edited the manuscript.

## Supplementary Material

Supplemental data

ICMJE disclosure forms

## Figures and Tables

**Table 1:**
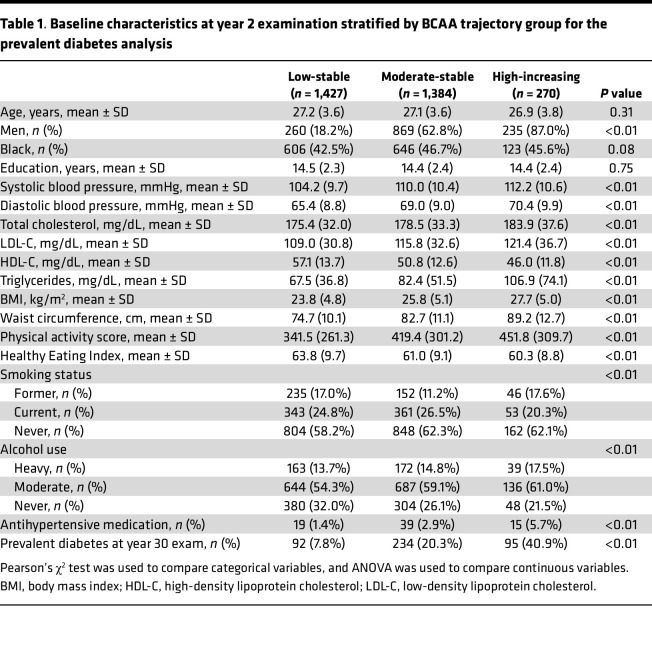
Baseline characteristics at year 2 examination stratified by BCAA trajectory group for the prevalent diabetes analysis

**Table 2:**
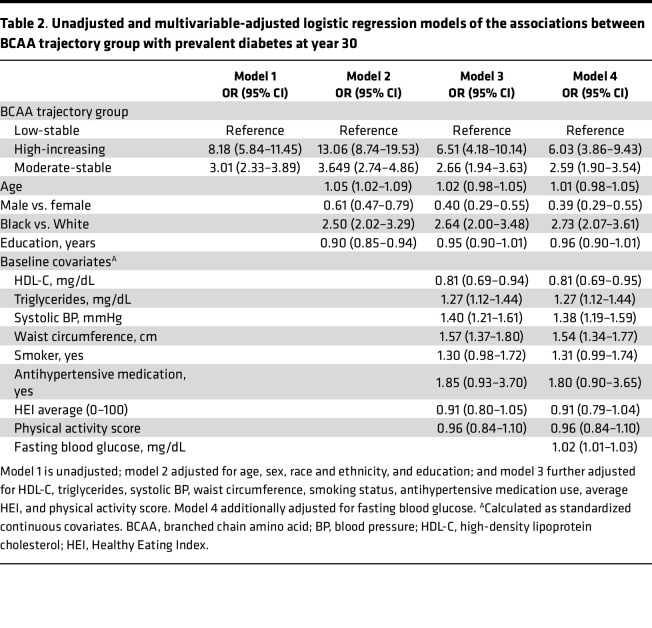
Unadjusted and multivariable-adjusted logistic regression models of the associations between BCAA trajectory group with prevalent diabetes at year 30

**Table 3:**
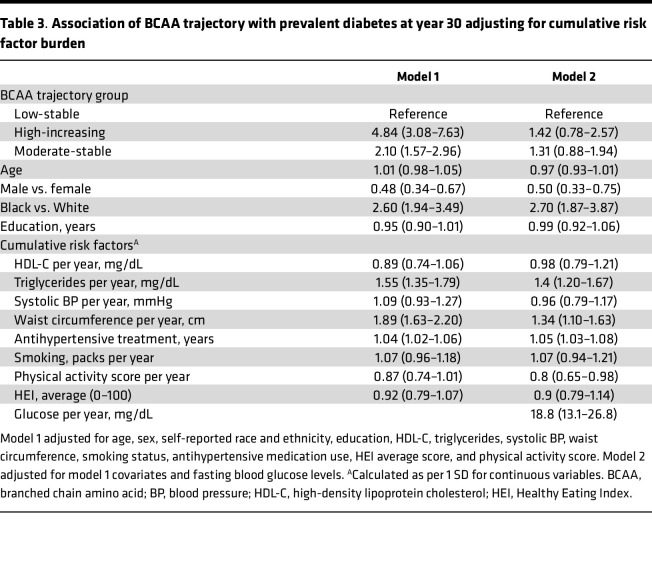
Association of BCAA trajectory with prevalent diabetes at year 30 adjusting for cumulative risk factor burden

**Table 4:**
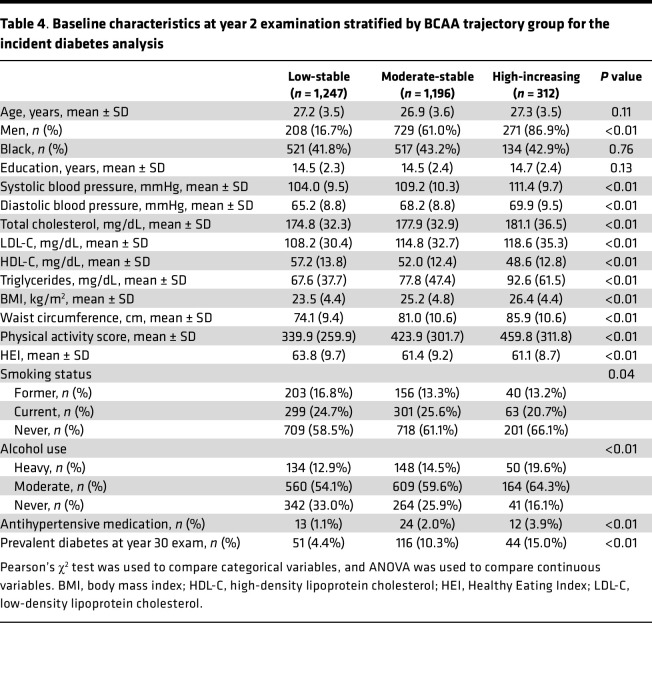
Baseline characteristics at year 2 examination stratified by BCAA trajectory group for the incident diabetes analysis

**Table 5:**
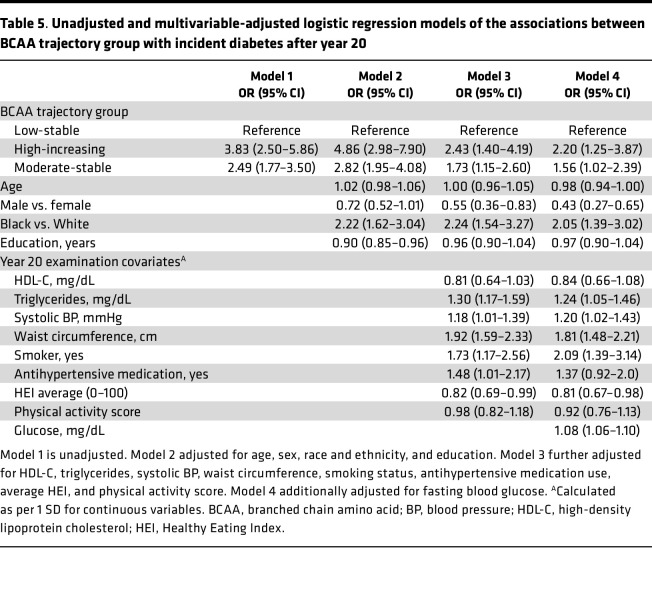
Unadjusted and multivariable-adjusted logistic regression models of the associations between BCAA trajectory group with incident diabetes after year 20

**Table 6:**
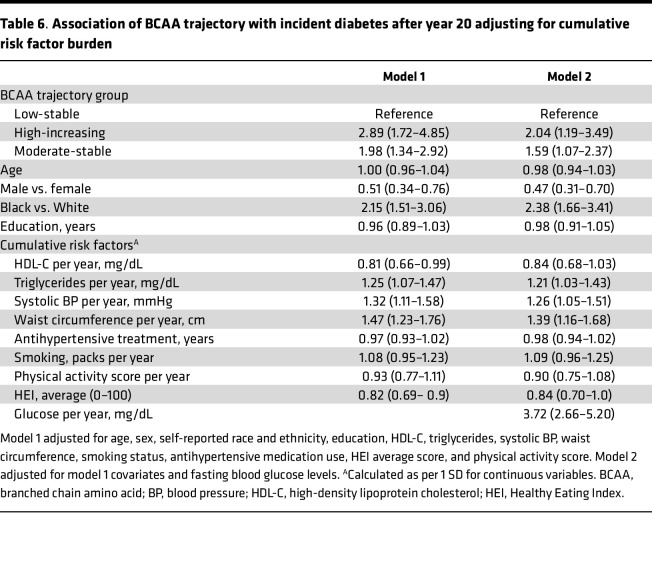
Association of BCAA trajectory with incident diabetes after year 20 adjusting for cumulative risk factor burden

## Version 1 04/24/2023

Electronic publication
