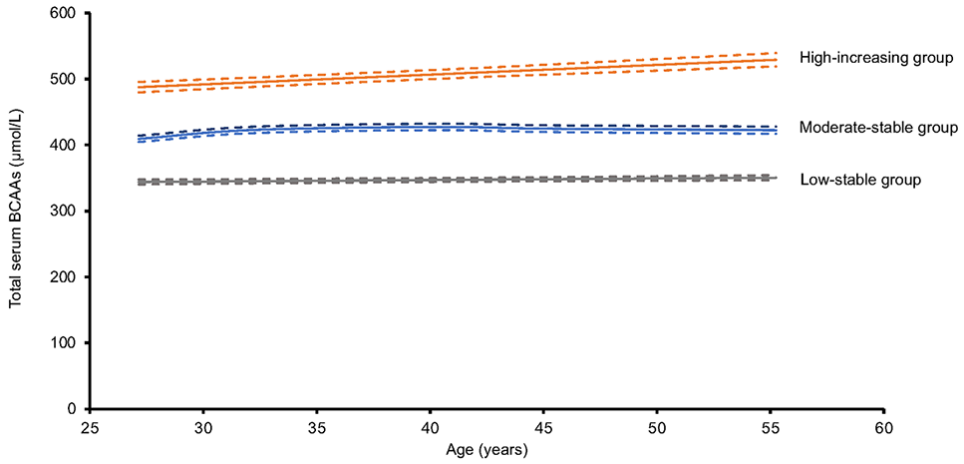# Longitudinal trajectories of branched chain amino acids through young adulthood and diabetes in later life

**DOI:** 10.1172/jci.insight.181901

**Published:** 2024-06-10

**Authors:** Konrad T. Sawicki, Hongyan Ning, Norrina B. Allen, Mercedes R. Carnethon, Amisha Wallia, James D. Otvos, Issam Ben-Sahra, Elizabeth M. McNally, Janet K. Snell-Bergeon, John T. Wilkins

## Abstract

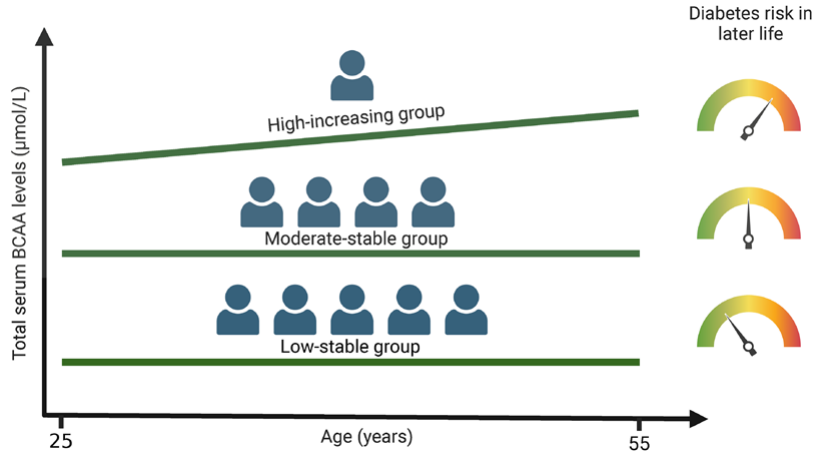

Original citation *JCI Insight*. 2023;8(8):e166956. https://doi.org/10.1172/jci.insight.166956

Citation for this corrigendum: *JCI Insight*. 2024;9(10):e181901. https://doi.org/10.1172/jci.insight.181901

The authors were recently notified by a reader of a potential error in the units for branched-chain amino acids. After review, the authors confirmed that the reported units (mg/dL) in the Results section, Graphical Abstract, Figures 2 and 3, and Supplemental Tables 1, 2, 4, and 5 were incorrect. The correct units are µmol/L. The corrected section of the Results section, the updated Graphical Abstract, and correct versions of Figures 2 and 3 appear below. The supplemental material file has been updated with the correct versions of Supplemental Tables 1, 2, 4, and 5. The HTML and PDF versions of the article have been updated online.

The authors regret the error.

Results

Longitudinal trajectories of circulating BCAAs from the year 2 to 30 exams.

The annualized rate of change in mean total BCAA levels from year 2 to year 30 examinations by trajectory group was 0.5 µmol/L/y in the low-stable group, 0.9 µmol/L/y in the moderate-stable group, and 2.3 µmol/L/y in the high-increasing group (Supplemental Table 4).

Association of longitudinal BCAA trajectories with incident DM at year 30.

The annualized rate of change in total BCAA levels from year 2 to year 30 examinations by trajectory group was 0.8 µmol/L in the low-stable group, 0.9 µmol/L in the moderate-stable group, and 1.9 µmol/L in the high-increasing group (Supplemental Table 5).

## Supplementary Material

Supplemental data

Supplemental tables 1-5

## Figures and Tables

**Figure 3:**
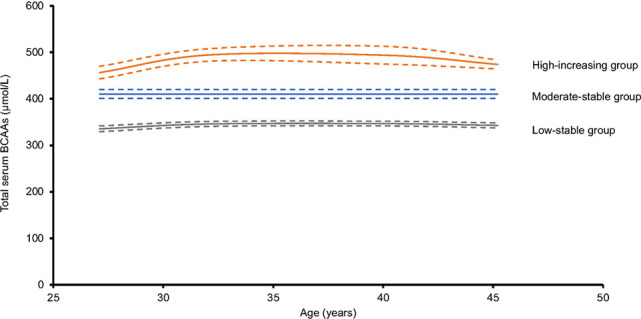


**Figure 2:**