# Supplementary material for: Longitudinal trajectories of branched chain amino acids through young adulthood and diabetes in later life
Source: JCI Insight. 2023 Apr 24;8(8):e166956. doi: 10.1172/jci.insight.166956 (PMC10243737; doi:10.1172/jci.insight.166956)
Supplement: Supplemental data [file jciinsight-8-166956-s079.pdf]

SUPPLEMENTAL MATERIAL

SUPPLEMENTAL TABLES

Supplemental Table 1. Baseline characteristics at Year 2 examination stratified by quartile of initial BCAA measurement.

	Quartile 1 (n=777)	Quartile 2 (n=778)	Quartile 3 (n=778)	Quartile 4 (n=778)	Total (n=3,111)
BCAA levels, mg/dL, mean ± SD					
Year 2 Examination	284.4 (33.0)	348.5 (13.5)	395.9 (14.8)	473.5 (52.7)	369.3 (75.6)
Year 7 Examination	362.4 (65.3)	393.4 (65.7)	420.9 (66.8)	475.0 (76.1)	413.4 (80.1)
Year 15 Examination	355.0 (63.8)	377.8 (67.8)	400.0 (67.6)	436.6 (77.5)	392.8 (75.6)
Year 20 Examination	359.2 (66.1)	384.5 (73.1)	406.2 (70.5)	438.5 (76.2)	397.0 (77.2)
Age, years, mean ± SD	27.1 (3.6)	27.1 (3.6)	27.3 (3.4)	27.1 (3.6)	27.2 (3.6)
Men, n (%)	169 (21.8%)	284 (36.5%)	396 (50.9%)	527 (67.7%)	1376 (44.2%)
Black, n (%)	346 (44.5%)	335 (43.1%)	338 (43.4%)	372 (47.8%)	1391 (44.7%)
Education, years, mean ± SD	14.3 (2.3)	14.5 (2.3)	14.5 (2.3)	14.4 (2.5)	14.4 (2.3)
Total cholesterol, mg/dL, mean ± SD	172.8 (32.4)	176.6 (32.3)	179.6 (33.5)	181.4 (34.3)	177.6 (33.3)
LDL-C, mg/dL, mean ± SD	106.7 (30.8)	111.9 (31.3)	116.0 (32.6)	117.9 (34.3)	113.1 (32.5)
HDL-C, mg/dL, mean ± SD	56.5 (13.7)	54.8 (13.1)	52.1 (13.6)	49.4 (12.8)	53.2 (13.6)
Triglycerides, mg/dL, mean ± SD	68.7 (39.0)	70.9 (42.3)	79.6 (46.2)	93.6 (75.6)	78.1 (53.5)
BMI, kg/m^2, mean ± SD	23.6 (4.6)	24.4 (4.6)	25.4 (5.0)	26.9 (5.6)	25.1 (5.1)
Fasting blood glucose, mg/dL, mean ± SD	81.0 (9.5)	84.3 (11.5)	86.0 (13.1)	91.3 920.4)	85.3 (14.3)
Systolic blood pressure, mmHg, mean ± SD	104.7 (9.8)	106.1 (10.6)	108.5 (10.1)	111.1 (10.5)	107.6 (10.6)
Diastolic blood pressure, mmHg, mean ± SD	65.7 (8.8)	66.3 (9.1)	68.1 (9.1)	69.9 (9.4)	67.5 (9.2)
Physical activity score, mean ± SD	360.5 (285.9)	377.7 (270.0)	392.0 (297.1)	412.3 (294.7)	385.5 (287.6)
Healthy Eating Index, mean ± SD	63.3 (9.7)	63.3 (9.3)	61.5 (9.5)	60.9 (8.9)	62.2 (9.4)
Smoking status					
Former, n (%)	115 (15.0%)	121 (16.0%)	105 (13.9%)	96 (12.7%)	437 (14.4%)
Current, n (%)	199 (25.9%)	179 (23.7%)	192 (25.4%)	198 (26.3%)	768 (25.3%)
Never, n (%)	463 (59.6%)	478 (61.4%)	481 (61.8%)	484 (62.2%)	1906 (61.3%)
Alcohol use					
Heavy, n (%)	73 (11.2%)	92 (14.3%)	113 (17.2%)	100 (15.6%)	378 (14.6%)
Moderate, n (%)	359 (55.1%)	368 (57.1%)	385 (58.7%)	368 (57.3%)	1480 (57.0%)
Never, n (%)	345 (44.4%	318 (40.9%)	280 (36.0%)	310 (39.8%)	1253 (40.3%)
Anti-hypertensive medication, n (%)	12 (1.6%)	17 (2.2%)	22 (2.9%)	27 (3.6%)	78 (2.6%)
Prevalent diabetes at Year 2 Exam, n (%)	5 (0.7%)	6 (0.8%)	5 (0.7%)	13 (1.7%)	29 (1.0%)
Prevalent diabetes at Year 30 Exam, n (%)	60 (10%)	89 (13.5%)	106 (16.4%)	167 (25.5%)	422 (16.5%)

Abbreviations: BCAA, branched chain amino acid; BMI, body mass index; LDL-C, low-density lipoprotein cholesterol; HDL-C, high-density lipoprotein cholesterol; SD, standard deviation.

Supplemental Table 2. Baseline characteristics at Year 2 examination stratified by sex across quartile of initial BCAA measurement.

	Quartile 1	Quartile 2	Quartile 3	Quartile 4	Total
BCAA levels, mg/dL, mean ± SD					
Year 2 Examination					
Women	281.0 (34.3)	347.8 (13.3)	394.5 (15.0)	462.7 (45.5)	340.5 (66.5)
Men	295.0 (26.1)	349.5 (13.6)	396.8 (14.7)	477.0 (54.5)	398.7 (72.9)
Year 7 Examination					
Women	351.2 (62.4)	374.0 (56.1)	394.1 (52.4)	455.0 (73.4)	382.9 (69.3)
Men	402.2 (60.1)	426.7 (67.8)	447.1 (69.0)	484.9 (75.5)	451.8 (76.1)
Year 15 Examination					
Women	342.7 (56.7)	358.3 (58.2)	377.2 (61.1)	412.8 (79.3)	365.4 (66.2)
Men	397.3 (68.6)	411.8 (70.0)	422.8 (66.1)	448.2 (74.0)	427.2 (72.5)
Year 20 Examination					
Women	346.3 (57.7)	365.7 (61.4)	376.9 (58.9)	402.0 (70.3)	366.5 (63.7)
Men	406.4 (73.4)	417.5 (80.2)	434.8 (69.1)	456.1 (72.7)	435.9 (75.6)
Age, years, mean ± SD					
Women	27.2 (3.7)	27.1 (3.6)	27.1 (3.5)	27.0 (3.7)	27.1 (3.6)
Men	26.9 (3.5)	27.2 (3.6)	27.4 (3.3)	27.2 (3.6)	27.2 (3.5)
Sex, n (%)					
Women	608 (35.0%)	494 (28.5%)	382 (22.0%)	251 (14.5%)	1735 (100%)
Men	169 (12.3%)	284 (20.6%)	396 (28.8%)	527 (38.3%)	1376 (100%)
Black, n (%)					
Women	275 (45.2%)	229 (46.4%)	189 (49.5%)	143 (57.0%)	836 (48.2%)
Men	71 (42.0%)	106 (37.3%)	149 (37.6%)	229 (43.5%)	555 (40.3%)
Education, years, mean ± SD					
Women	14.4 (2.2)	14.5 (2.1)	14.4 (2.3)	14.2 (2.2)	14.4 (2.2)
Men	14.1 (2.4)	14.5 (2.6)	14.5 (2.3)	14.5 (2.6)	14.5 (2.5)
Total cholesterol, mg/dL, mean ± SD					
Women	173.5 (31.5)	178.2 (30.7)	178.2 (30.7)	179.7 (34.4)	179.2 (30.5)
Men	170.6 (35.5)	173.9 (34.8)	179.5 (32.6)	182.4 (35.9)	178.3 (34.9)
LDL-C, mg/dL, mean ± SD					
Women	106.1 (29.6)	111.7 (29.7)	113.9 (33.4)	113.9 (30.8)	110.5 (30.8)
Men	108.9 (35.0)	112.1 (33.8)	118.0 (31.8)	119.8 (35.6)	116.3 (34.3)
HDL-C, mg/dL, mean ± SD					
Women	58.0 (13.7)	57.2 (13.2)	55.9 (13.6)	53.5 (12.0)	56.7 (13.4)
Men	51.0 (12.1)	50.9 (12.0)	48.6 (12.6)	47.5 (12.7)	49.0 (12.5)
Triglycerides, mg/dL, mean ± SD					
Women	67.1 (36.1)	67.9 (38.0)	71.5 (38.8)	81.3 (82.2)	70.3 (46.6)
Men	74.5 (47.4)	76.0 (48.3)	87.2 (51.0)	99.3 (71.7)	87.9 (59.6)
BMI, kg/m^2, mean ± SD					
Women	23.8 (4.9)	24.5 (5.2)	25.9 (6.1)	27.6 (6.9)	25.0 (5.7)
Men	23.2 (2.9)	24.3 (3.3)	25.0 (3.8)	26.5 (4.8)	25.2 (4.2)
Fasting blood glucose, mg/dL, mean ± SD					
Women	80.5 (9.7)	83.4 (12.7)	86.0 (17.4)	93.6 (28.6)	83.7 (15.6)
Men	82.8 (8.9)	85.4 (9.8)	85.9 (9.4)	90.6 (16.9)	86.9 (12.7)
Systolic blood pressure, mmHg, mean ± SD					
Women	103.0 (9.2)	102.9 (9.3)	104.6 (9.3)	106.6 (9.6)	103.8 (9.4)
Men	110.9 (9.6)	111.5 (10.6)	112.3 (9.3)	113.3 (10.3)	112.3 (10.0)
Diastolic blood pressure, mmHg, mean ± SD					
Women	65.0 (8.6)	64.8 (8.7)	65.8 (9.3)	67.8 (9.0)	65.5 (8.9)

Men	68.6 (8.8)	69.0 (9.3)	70.2 (8.4)	70.9 (9.4)	70.0 (9.0)
Physical activity score, mean ± SD					
Women	308.7 (241.9)	323.3 (232.2)	298.2 (240.1)	276.2 (228.0)	305.9 (237.1)
Men	545.1 (348.4)	471.3 (303.5)	482.1 (318.3)	477.1 (300.9)	485.9 (313.2)
Healthy Eating Index, mean ± SD					
Women	63.7 (9.8)	64.3 (9.2)	62.8 (9.6)	62.6 (9.1)	63.5 (9.5)
Men	61.8 (9.1)	61.5 (9.4)	60.2 (9.3)	60.2 (8.8)	60.6 (9.1)
Smoking status					
Former, n (%)					
Women	98 (16.3%)	76 (16.0%)	55 (14.9%)	36 (14.7%)	265 (15.7%)
Men	17 (10.2%)	45 (16.2%)	50 (12.9%)	60 (11.8%)	172 (12.8%)
Current, n (%)					
Women	150 (25.0%)	105 (22.1%)	92 (24.9%)	67 (27.3%)	414 (24.5%)
Men	49 (29.3%)	74 (26.6%)	100 (25.8%)	131 (25.8%)	354 (26.4%)
Never, n (%)					
Women	353 (58.7%)	295 (62.0%)	223 (60.3%)	142 (58.0%)	1013 (59.9%)
Men	101 (60.5%)	159 (57.2%)	237 (61.2%)	317 (62.4%)	814 (60.7%)
Alcohol use					
Heavy, n (%)					
Women	61 (12.2%)	54 (13.5%)	56 (18.4%)	31 (15.5%)	202 (14.4%)
Men	12 (7.9%)	38 (15.5%)	57 (16.2%)	69 (15.6%)	176 (14.8%)
Moderate, n (%)					
Women	250 (49.9%)	207 (51.8%)	142 (46.7%)	85 (42.5%)	684 (48.7%)
Men	109 (72.2%)	161 (65.7%)	243 (69.0%)	283 (64.0%)	796 (66.9%)
Never, n (%)					
Women	190 (37.9%)	139 (34.8%)	106 (34.9%)	84 (42.0%)	519 (36.9%)
Men	30 (19.9%)	46 (18.8%)	52 (14.8%)	90 (20.4%)	218 (18.3%)
Anti-hypertensive medication, n (%)					
Women	12 (2.0%)	12 (2.5%)	12 (3.2%)	10 (4.1%)	46 (2.7%)
Men	0 (0.0%)	5 (1.8%)	10 (2.6%)	17 (3.3%)	32 (2.4%)
Prevalent diabetes at Year 2 Exam, n (%)					
Women	2 (0.3%)	1 (0.2%)	4 (1.1%)	5 (2.0%)	12 (0.7%)
Men	1 (0.6%)	2 (0.7%)	0 (0.0%)	2 (0.4%)	5 (0.4%)
Prevalent diabetes at Year 30 Exam, n (%)					
Women	44 (9.4%)	55 (13.1%)	55 (17.5%)	59 (27.7%)	213 (15.1%)
Men	16 (11.9%)	34 (14.4%)	51 (15.3%)	108 (24.4%)	209 (18.2%)

Abbreviations: BCAA, branched chain amino acid; BMI, body mass index; LDL-C, low-density lipoprotein cholesterol; HDL-C, high-density lipoprotein cholesterol; SD, standard deviation.

Supplemental Table 3. Clinical characteristics at Year 2 Examination by study inclusion status.

	**Excluded (n=2,031)**	**Included (n=3,081)**
Age, years, mean ± SD	26.6 (3.7)	27.1 (3.6)
Men, n (%)	963 (47.4%)	1364 (44.3%)
Black, n (%)	1262 (62.1%)	1375 (44.6%)
Education, years, mean ± SD	13.7 (3.8)	14.4 (2.3)
Systolic blood pressure, mmHg, mean ± SD	108.4 (11.2)	107.6 (10.6)
Diastolic blood pressure, mmHg, mean ± SD	67.3 (10.5)	67.5 (9.2)
Total cholesterol, mg/dL, mean ± SD	175.8 (35.6)	177.6 (33.2)
HDL-C, mg/dL, mean ± SD	52.6 (13.9)	53.3 (13.6)
Triglycerides, mg/dL, mean ± SD	81.3 (60.5)	77.7 (49.3)
BMI, kg/m^2, mean ± SD	25.5 (5.9)	25.1 (5.1)
Waist circumference, cm, mean ± SD	80.5 (12.9)	79.6 (11.9)
Physical activity score, mean ± SD	373.9 (291.5)	386.3 (287.3)
Healthy Eating Index, mean ± SD	62.3 (9.8)	62.2 (9.4)
Smoking status		
Former, n (%)	192 (12.1%)	433 (14.4%)
Current, n (%)	601 (37.8%)	757 (25.2%)
Never, n (%)	798 (50.2%)	1814 (60.4%)
Alcohol use		
Heavy, n (%)	4 (18.2%)	374 (14.5%)
Moderate, n (%)	13 (59.1%)	1467 (57.0%)
Never, n (%)	5 (22.7%)	732 (28.4%)
Anti-hypertensive medication, n (%)	50 (3.2%)	73 (2.4%)

Abbreviations: BMI, body mass index; HDL-C, high-density lipoprotein cholesterol; SD, standard deviation.

Supplemental Table 4. Annualized rates of change by BCAA trajectory group in prevalent diabetes analysis.

	**Low-stable (n=1,427)**	**Moderate-stable (n=1,384)**	**High-increasing (n=270)**
BCAA levels, mg/dL, mean ± SD			
Year 2 Examination	323.3 (56.8)	393.5 (61.1)	462.6 (77.4)
Year 7 Examination	361.2 (51.0)	442.4 (60.0)	535.7 (83.3)
Year 15 Examination	341.3 (46.8)	423.8 (56.5)	504.2 (75.8)
Year 20 Examination	344.3 (47.4)	428.1 (54.5)	517.8 (81.6)
Year 30 Examination	347.4 (53.5)	426.0 (57.1)	535.0 (86.9)
Annualized BCAA change	0.5 (3.3)	0.9 (4.0)	2.3 (6.7)

Abbreviations: BCAA, branched chain amino acid; SD, standard deviation.

Supplemental Table 5. Annualized rates of change by BCAA trajectory group in incident diabetes analysis.

	**Low-stable (n=1,247)**	**Moderate-stable (n=1,196)**	**High-increasing (n=312)**
BCAA levels, mg/dL, mean ± SD			
Year 2 Examination	314.6 (51.2)	392.9 (56.5)	450.6 (81.9)
Year 7 Examination	356.2 (48.3)	432.5 (50.9)	520.5 (76.0)
Year 15 Examination	338.7 (47.0)	411.3 (52.0)	493.2 (69.3)
Year 20 Examination	341.0 (46.5)	416.7 (51.7)	497.4 (77.9)
Annualized BCAA change	0.8 (4.4)	0.9 (5.0)	1.9 (7.8)

Abbreviations: BCAA, branched chain amino acid; SD, standard deviation.

Supplemental Table 6. Participants with available BCAA measurements by examination Year.

Examination	Number of participants
Year 2	2334
Year 7	2920
Year 15	2798
Year 20	3054
Year 30	2562
