# Supplementary material for: Longitudinal trajectories of branched chain amino acids through young adulthood and diabetes in later life
Source: JCI Insight. 2024 Jun 10;9(11):e181901. doi: 10.1172/jci.insight.181901 (PMC11382874; doi:10.1172/jci.insight.181901)
Supplement: Supplemental tables 1-5 [file jciinsight-9-181901-s207.docx]

**SUPPLEMENTAL MATERIAL**

**SUPPLEMENTAL TABLES**

**Supplemental Table 1. Baseline characteristics at Year 2 examination stratified by quartile of initial BCAA measurement.**

|  | **Quartile 1**  **(n=777)** | **Quartile 2**  **(n=778)** | **Quartile 3**  **(n=778)** | **Quartile 4**  **(n=778)** | **Total**  **(n=3,111)** |
| --- | --- | --- | --- | --- | --- |
| BCAA levels, µmol/L, mean ± SD |  |  |  |  |  |
| Year 2 Examination | 284.4 (33.0) | 348.5 (13.5) | 395.9 (14.8) | 473.5 (52.7) | 369.3 (75.6) |
| Year 7 Examination | 362.4 (65.3) | 393.4 (65.7) | 420.9 (66.8) | 475.0 (76.1) | 413.4 (80.1) |
| Year 15 Examination | 355.0 (63.8) | 377.8 (67.8) | 400.0 (67.6) | 436.6 (77.5) | 392.8 (75.6) |
| Year 20 Examination | 359.2 (66.1) | 384.5 (73.1) | 406.2 (70.5) | 438.5 (76.2) | 397.0 (77.2) |
| Age, years, mean ± SD | 27.1 (3.6) | 27.1 (3.6) | 27.3 (3.4) | 27.1 (3.6) | 27.2 (3.6) |
| Men, n (%) | 169 (21.8%) | 284 (36.5%) | 396 (50.9%) | 527 (67.7%) | 1376 (44.2%) |
| Black, n (%) | 346 (44.5%) | 335 (43.1%) | 338 (43.4%) | 372 (47.8%) | 1391 (44.7%) |
| Education, years, mean ± SD | 14.3 (2.3) | 14.5 (2.3) | 14.5 (2.3) | 14.4 (2.5) | 14.4 (2.3) |
| Total cholesterol, mg/dL, mean ± SD | 172.8 (32.4) | 176.6 (32.3) | 179.6 (33.5) | 181.4 (34.3) | 177.6 (33.3) |
| LDL-C, mg/dL, mean ± SD | 106.7 (30.8) | 111.9 (31.3) | 116.0 (32.6) | 117.9 (34.3) | 113.1 (32.5) |
| HDL-C, mg/dL, mean ± SD | 56.5 (13.7) | 54.8 (13.1) | 52.1 (13.6) | 49.4 (12.8) | 53.2 (13.6) |
| Triglycerides, mg/dL, mean ± SD | 68.7 (39.0) | 70.9 (42.3) | 79.6 (46.2) | 93.6 (75.6) | 78.1 (53.5) |
| BMI, kg/m^2^, mean ± SD | 23.6 (4.6) | 24.4 (4.6) | 25.4 (5.0) | 26.9 (5.6) | 25.1 (5.1) |
| Fasting blood glucose, mg/dL, mean ± SD | 81.0 (9.5) | 84.3 (11.5) | 86.0 (13.1) | 91.3 920.4) | 85.3 (14.3) |
| Systolic blood pressure, mmHg, mean ± SD | 104.7 (9.8) | 106.1 (10.6) | 108.5 (10.1) | 111.1 (10.5) | 107.6 (10.6) |
| Diastolic blood pressure, mmHg, mean ± SD | 65.7 (8.8) | 66.3 (9.1) | 68.1 (9.1) | 69.9 (9.4) | 67.5 (9.2) |
| Physical activity score, mean ± SD | 360.5 (285.9) | 377.7 (270.0) | 392.0 (297.1) | 412.3 (294.7) | 385.5 (287.6) |
| Healthy Eating Index, mean ± SD | 63.3 (9.7) | 63.3 (9.3) | 61.5 (9.5) | 60.9 (8.9) | 62.2 (9.4) |
| Smoking status |  |  |  |  |  |
| Former, n (%) | 115 (15.0%) | 121 (16.0%) | 105 (13.9%) | 96 (12.7%) | 437 (14.4%) |
| Current, n (%) | 199 (25.9%) | 179 (23.7%) | 192 (25.4%) | 198 (26.3%) | 768 (25.3%) |
| Never, n (%) | 463 (59.6%) | 478 (61.4%) | 481 (61.8%) | 484 (62.2%) | 1906 (61.3%) |
| Alcohol use |  |  |  |  |  |
| Heavy, n (%) | 73 (11.2%) | 92 (14.3%) | 113 (17.2%) | 100 (15.6%) | 378 (14.6%) |
| Moderate, n (%) | 359 (55.1%) | 368 (57.1%) | 385 (58.7%) | 368 (57.3%) | 1480 (57.0%) |
| Never, n (%) | 345 (44.4% | 318 (40.9%) | 280 (36.0%) | 310 (39.8%) | 1253 (40.3%) |
| Anti-hypertensive medication, n (%) | 12 (1.6%) | 17 (2.2%) | 22 (2.9%) | 27 (3.6%) | 78 (2.6%) |
| Prevalent diabetes at Year 2 Exam, n (%) | 5 (0.7%) | 6 (0.8%) | 5 (0.7%) | 13 (1.7%) | 29 (1.0%) |
| Prevalent diabetes at Year 30 Exam, n (%) | 60 (10%) | 89 (13.5%) | 106 (16.4%) | 167 (25.5%) | 422 (16.5%) |

Abbreviations: BCAA, branched chain amino acid; BMI, body mass index; LDL-C, low-density lipoprotein cholesterol; HDL-C, high-density lipoprotein cholesterol; SD, standard deviation.

**Supplemental Table 2. Baseline characteristics at Year 2 examination stratified by sex across quartile of initial BCAA measurement.**

|  | **Quartile 1** | **Quartile 2** | **Quartile 3** | **Quartile 4** | **Total** |
| --- | --- | --- | --- | --- | --- |
| BCAA levels, µmol/L, mean ± SD |  |  |  |  |  |
| Year 2 Examination |  |  |  |  |  |
| Women | 281.0 (34.3) | 347.8 (13.3) | 394.5 (15.0) | 462.7 (45.5) | 340.5 (66.5) |
| Men | 295.0 (26.1) | 349.5 (13.6) | 396.8 (14.7) | 477.0 (54.5) | 398.7 (72.9) |
| Year 7 Examination |  |  |  |  |  |
| Women | 351.2 (62.4) | 374.0 (56.1) | 394.1 (52.4) | 455.0 (73.4) | 382.9 (69.3) |
| Men | 402.2 (60.1) | 426.7 (67.8) | 447.1 (69.0) | 484.9 (75.5) | 451.8 (76.1) |
| Year 15 Examination |  |  |  |  |  |
| Women | 342.7 (56.7) | 358.3 (58.2) | 377.2 (61.1) | 412.8 (79.3) | 365.4 (66.2) |
| Men | 397.3 (68.6) | 411.8 (70.0) | 422.8 (66.1) | 448.2 (74.0) | 427.2 (72.5) |
| Year 20 Examination |  |  |  |  |  |
| Women | 346.3 (57.7) | 365.7 (61.4) | 376.9 (58.9) | 402.0 (70.3) | 366.5 (63.7) |
| Men | 406.4 (73.4) | 417.5 (80.2) | 434.8 (69.1) | 456.1 (72.7) | 435.9 (75.6) |
| Age, years, mean ± SD |  |  |  |  |  |
| Women | 27.2 (3.7) | 27.1 (3.6) | 27.1 (3.5) | 27.0 (3.7) | 27.1 (3.6) |
| Men | 26.9 (3.5) | 27.2 (3.6) | 27.4 (3.3) | 27.2 (3.6) | 27.2 (3.5) |
| Sex, n (%) |  |  |  |  |  |
| Women | 608 (35.0%) | 494 (28.5%) | 382 (22.0%) | 251 (14.5%) | 1735 (100%) |
| Men | 169 (12.3%) | 284 (20.6%) | 396 (28.8%) | 527 (38.3%) | 1376 (100%) |
| Black, n (%) |  |  |  |  |  |
| Women | 275 (45.2%) | 229 (46.4%) | 189 (49.5%) | 143 (57.0%) | 836 (48.2%) |
| Men | 71 (42.0%) | 106 (37.3%) | 149 (37.6%) | 229 (43.5%) | 555 (40.3%) |
| Education, years, mean ± SD |  |  |  |  |  |
| Women | 14.4 (2.2) | 14.5 (2.1) | 14.4 (2.3) | 14.2 (2.2) | 14.4 (2.2) |
| Men | 14.1 (2.4) | 14.5 (2.6) | 14.5 (2.3) | 14.5 (2.6) | 14.5 (2.5) |
| Total cholesterol, mg/dL, mean ± SD |  |  |  |  |  |
| Women | 173.5 (31.5) | 178.2 (30.7) | 178.2 (30.7) | 179.7 (34.4) | 179.2 (30.5) |
| Men | 170.6 (35.5) | 173.9 (34.8) | 179.5 (32.6) | 182.4 (35.9) | 178.3 (34.9) |
| LDL-C, mg/dL, mean ± SD |  |  |  |  |  |
| Women | 106.1 (29.6) | 111.7 (29.7) | 113.9 (33.4) | 113.9 (30.8) | 110.5 (30.8) |
| Men | 108.9 (35.0) | 112.1 (33.8) | 118.0 (31.8) | 119.8 (35.6) | 116.3 (34.3) |
| HDL-C, mg/dL, mean ± SD |  |  |  |  |  |
| Women | 58.0 (13.7) | 57.2 (13.2) | 55.9 (13.6) | 53.5 (12.0) | 56.7 (13.4) |
| Men | 51.0 (12.1) | 50.9 (12.0) | 48.6 (12.6) | 47.5 (12.7) | 49.0 (12.5) |
| Triglycerides, mg/dL, mean ± SD |  |  |  |  |  |
| Women | 67.1 (36.1) | 67.9 (38.0) | 71.5 (38.8) | 81.3 (82.2) | 70.3 (46.6) |
| Men | 74.5 (47.4) | 76.0 (48.3) | 87.2 (51.0) | 99.3 (71.7) | 87.9 (59.6) |
| BMI, kg/m^2^, mean ± SD |  |  |  |  |  |
| Women | 23.8 (4.9) | 24.5 (5.2) | 25.9 (6.1) | 27.6 (6.9) | 25.0 (5.7) |
| Men | 23.2 (2.9) | 24.3 (3.3) | 25.0 (3.8) | 26.5 (4.8) | 25.2 (4.2) |
| Fasting blood glucose, mg/dL, mean ± SD |  |  |  |  |  |
| Women | 80.5 (9.7) | 83.4 (12.7) | 86.0 (17.4) | 93.6 (28.6) | 83.7 (15.6) |
| Men | 82.8 (8.9) | 85.4 (9.8) | 85.9 (9.4) | 90.6 (16.9) | 86.9 (12.7) |
| Systolic blood pressure, mmHg, mean ± SD |  |  |  |  |  |
| Women | 103.0 (9.2) | 102.9 (9.3) | 104.6 (9.3) | 106.6 (9.6) | 103.8 (9.4) |
| Men | 110.9 (9.6) | 111.5 (10.6) | 112.3 (9.3) | 113.3 (10.3) | 112.3 (10.0) |
| Diastolic blood pressure, mmHg, mean ± SD |  |  |  |  |  |
| Women | 65.0 (8.6) | 64.8 (8.7) | 65.8 (9.3) | 67.8 (9.0) | 65.5 (8.9) |
| Men | 68.6 (8.8) | 69.0 (9.3) | 70.2 (8.4) | 70.9 (9.4) | 70.0 (9.0) |
| Physical activity score, mean ± SD |  |  |  |  |  |
| Women | 308.7 (241.9) | 323.3 (232.2) | 298.2 (240.1) | 276.2 (228.0) | 305.9 (237.1) |
| Men | 545.1 (348.4) | 471.3 (303.5) | 482.1 (318.3) | 477.1 (300.9) | 485.9 (313.2) |
| Healthy Eating Index, mean ± SD |  |  |  |  |  |
| Women | 63.7 (9.8) | 64.3 (9.2) | 62.8 (9.6) | 62.6 (9.1) | 63.5 (9.5) |
| Men | 61.8 (9.1) | 61.5 (9.4) | 60.2 (9.3) | 60.2 (8.8) | 60.6 (9.1) |
| Smoking status |  |  |  |  |  |
| Former, n (%) |  |  |  |  |  |
| Women | 98 (16.3%) | 76 (16.0%) | 55 (14.9%) | 36 (14.7%) | 265 (15.7%) |
| Men | 17 (10.2%) | 45 (16.2%) | 50 (12.9%) | 60 (11.8%) | 172 (12.8%) |
| Current, n (%) |  |  |  |  |  |
| Women | 150 (25.0%) | 105 (22.1%) | 92 (24.9%) | 67 (27.3%) | 414 (24.5%) |
| Men | 49 (29.3%) | 74 (26.6%) | 100 (25.8%) | 131 (25.8%) | 354 (26.4%) |
| Never, n (%) |  |  |  |  |  |
| Women | 353 (58.7%) | 295 (62.0%) | 223 (60.3%) | 142 (58.0%) | 1013 (59.9%) |
| Men | 101 (60.5%) | 159 (57.2%) | 237 (61.2%) | 317 (62.4%) | 814 (60.7%) |
| Alcohol use |  |  |  |  |  |
| Heavy, n (%) |  |  |  |  |  |
| Women | 61 (12.2%) | 54 (13.5%) | 56 (18.4%) | 31 (15.5%) | 202 (14.4%) |
| Men | 12 (7.9%) | 38 (15.5%) | 57 (16.2%) | 69 (15.6%) | 176 (14.8%) |
| Moderate, n (%) |  |  |  |  |  |
| Women | 250 (49.9%) | 207 (51.8%) | 142 (46.7%) | 85 (42.5%) | 684 (48.7%) |
| Men | 109 (72.2%) | 161 (65.7%) | 243 (69.0%) | 283 (64.0%) | 796 (66.9%) |
| Never, n (%) |  |  |  |  |  |
| Women | 190 (37.9%) | 139 (34.8%) | 106 (34.9%) | 84 (42.0%) | 519 (36.9%) |
| Men | 30 (19.9%) | 46 (18.8%) | 52 (14.8%) | 90 (20.4%) | 218 (18.3%) |
| Anti-hypertensive medication, n (%) |  |  |  |  |  |
| Women | 12 (2.0%) | 12 (2.5%) | 12 (3.2%) | 10 (4.1%) | 46 (2.7%) |
| Men | 0 (0.0%) | 5 (1.8%) | 10 (2.6%) | 17 (3.3%) | 32 (2.4%) |
| Prevalent diabetes at Year 2 Exam, n (%) |  |  |  |  |  |
| Women | 2 (0.3%) | 1 (0.2%) | 4 (1.1%) | 5 (2.0%) | 12 (0.7%) |
| Men | 1 (0.6%) | 2 (0.7%) | 0 (0.0%) | 2 (0.4%) | 5 (0.4%) |
| Prevalent diabetes at Year 30 Exam, n (%) |  |  |  |  |  |
| Women | 44 (9.4%) | 55 (13.1%) | 55 (17.5%) | 59 (27.7%) | 213 (15.1%) |
| Men | 16 (11.9%) | 34 (14.4%) | 51 (15.3%) | 108 (24.4%) | 209 (18.2%) |

Abbreviations: BCAA, branched chain amino acid; BMI, body mass index; LDL-C, low-density lipoprotein cholesterol; HDL-C, high-density lipoprotein cholesterol; SD, standard deviation.

**Supplemental Table 3. Clinical characteristics at Year 2 Examination by study inclusion status.**

|  | **Excluded**  **(n=2,031)** | **Included**  **(n=3,081)** |
| --- | --- | --- |
| Age, years, mean ± SD | 26.6 (3.7) | 27.1 (3.6) |
| Men, n (%) | 963 (47.4%) | 1364 (44.3%) |
| Black, n (%) | 1262 (62.1%) | 1375 (44.6%) |
| Education, years, mean ± SD | 13.7 (3.8) | 14.4 (2.3) |
| Systolic blood pressure, mmHg, mean ± SD | 108.4 (11.2) | 107.6 (10.6) |
| Diastolic blood pressure, mmHg, mean ± SD | 67.3 (10.5) | 67.5 (9.2) |
| Total cholesterol, mg/dL, mean ± SD | 175.8 (35.6) | 177.6 (33.2) |
| HDL-C, mg/dL, mean ± SD | 52.6 (13.9) | 53.3 (13.6) |
| Triglycerides, mg/dL, mean ± SD | 81.3 (60.5) | 77.7 (49.3) |
| BMI, kg/m^2^, mean ± SD | 25.5 (5.9) | 25.1 (5.1) |
| Waist circumference, cm, mean ± SD | 80.5 (12.9) | 79.6 (11.9) |
| Physical activity score, mean ± SD | 373.9 (291.5) | 386.3 (287.3) |
| Healthy Eating Index, mean ± SD | 62.3 (9.8) | 62.2 (9.4) |
| Smoking status |  |  |
| Former, n (%) | 192 (12.1%) | 433 (14.4%) |
| Current, n (%) | 601 (37.8%) | 757 (25.2%) |
| Never, n (%) | 798 (50.2%) | 1814 (60.4%) |
| Alcohol use |  |  |
| Heavy, n (%) | 4 (18.2%) | 374 (14.5%) |
| Moderate, n (%) | 13 (59.1%) | 1467 (57.0%) |
| Never, n (%) | 5 (22.7%) | 732 (28.4%) |
| Anti-hypertensive medication, n (%) | 50 (3.2%) | 73 (2.4%) |

Abbreviations: BMI, body mass index; HDL-C, high-density lipoprotein cholesterol; SD, standard deviation.

**Supplemental Table 4. Annualized rates of change by BCAA trajectory group in prevalent diabetes analysis.**

|  | **Low-stable**  **(n=1,427)** | **Moderate-stable**  **(n=1,384)** | **High-increasing**  **(n=270)** |
| --- | --- | --- | --- |
| BCAA levels, µmol/L, mean ± SD |  |  |  |
| Year 2 Examination | 323.3 (56.8) | 393.5 (61.1) | 462.6 (77.4) |
| Year 7 Examination | 361.2 (51.0) | 442.4 (60.0) | 535.7 (83.3) |
| Year 15 Examination | 341.3 (46.8) | 423.8 (56.5) | 504.2 (75.8) |
| Year 20 Examination | 344.3 (47.4) | 428.1 (54.5) | 517.8 (81.6) |
| Year 30 Examination | 347.4 (53.5) | 426.0 (57.1) | 535.0 (86.9) |
| Annualized BCAA change | 0.5 (3.3) | 0.9 (4.0) | 2.3 (6.7) |

Abbreviations: BCAA, branched chain amino acid; SD, standard deviation.

**Supplemental Table 5. Annualized rates of change by BCAA trajectory group in incident diabetes analysis.**

|  | **Low-stable**  **(n=1,247)** | **Moderate-stable**  **(n=1,196)** | **High-increasing**  **(n=312)** |
| --- | --- | --- | --- |
| BCAA levels, µmol/L, mean ± SD |  |  |  |
| Year 2 Examination | 314.6 (51.2) | 392.9 (56.5) | 450.6 (81.9) |
| Year 7 Examination | 356.2 (48.3) | 432.5 (50.9) | 520.5 (76.0) |
| Year 15 Examination | 338.7 (47.0) | 411.3 (52.0) | 493.2 (69.3) |
| Year 20 Examination | 341.0 (46.5) | 416.7 (51.7) | 497.4 (77.9) |
| Annualized BCAA change | 0.8 (4.4) | 0.9 (5.0) | 1.9 (7.8) |

Abbreviations: BCAA, branched chain amino acid; SD, standard deviation.

**Supplemental Table 6. Participants with available BCAA measurements by examination Year.**

| **Examination** | **Number of participants** |
| --- | --- |
| Year 2 | 2334 |
| Year 7 | 2920 |
| Year 15 | 2798 |
| Year 20 | 3054 |
| Year 30 | 2562 |
